# Colour vision of green turtle (*Chelonia mydas*) hatchlings: do they still prefer blue under water?

**DOI:** 10.7717/peerj.5572

**Published:** 2018-09-19

**Authors:** Rebecca Jehne Hall, Simon K.A. Robson, Ellen Ariel

**Affiliations:** 1 College of Science and Engineering: Zoology and Ecology, James Cook University, Townsville, Queensland, Australia; 2 College of Public Health, Medical and Veterinary Sciences, James Cook University, Townsville, Queensland, Australia

**Keywords:** Marine turtle colour vision, Species-specific management tools, Innate behavioural attraction, Colour vision management, Green turtle management

## Abstract

**Background:**

Several anatomical studies provide evidence that green turtles (*Chelonia mydas)* possess the necessary anatomy for colour vision. Behavioural experiments have previously been conducted with newly emerged hatchlings, concluding that they are attracted to shorter wavelengths compared to longer wavelengths within a terrestrial environment, suggesting a possible attraction towards blue. This paper assessed the colour vision of hatchlings within an aquatic environment, and investigated whether the attraction for shorter wavelengths remains consistent within water, whether the colour saturation of the chromatic stimuli was an important factor, and whether rearing and testing individual animals in different coloured housing tanks has an impact on their visual choices.

**Methods:**

Forty-one hatchling green turtles were presented with a three-choice experiment where food was attached to three different coloured plates. The plates (blue, yellow, and red) were randomly arranged in the turtle’s tank and four different colour saturations were tested (100, 75, 50, and 25%). Turtles were individually placed into their housing tanks (coloured either red, white, blue or grey) with three different colour plates in front of them, from the same saturation level. The colour of the plate with food first approached and bitten by the turtle was recorded.

**Results:**

The colour of the tank in which an individual was reared, and where experiments were conducted, significantly influenced which food item was selected on the different coloured plates. While individual turtles preferred to select the food items associated with blue plates across the entire experiment (66.1% of the time compared to 18.2% and 15.7% for yellow and red plates respectively), the preference for blue plates was influenced by the colour of the rearing/experimental tank. Individuals raised in red, white or blue tanks appeared to consistently prefer food on blue plates, but there appeared to be no plate colour preference by turtles in grey tanks. There was no significant effect of either colour saturation or the spatial arrangement of the three colours within an experimental tank on colour choice, and no significant interaction between tank colour and colour saturation.

**Discussion:**

These****findings confirm that the terrestrial preference towards shorter wavelength colours, such as blue, compared to longer wavelength colours remains consistent within an aquatic environment. This preference for blue continues even as the colour saturation reduces from 100% down to 25%, and the colours become darker. Thus, it is suggested that green turtle hatchlings have a strong attraction towards blue. This attraction, however, is influenced by the colour of the tank the turtles were raised in. While this supports the notion that environmental colour may influence individual turtle visual capabilities, it suggests that this relationship is more complicated, and requires further investigation.

## Introduction

Colour vision has been documented in numerous animals, and it forms an integral part of receiving crucial colour signals from other animals and the environment. Certain birds, for example, have remarkable colour displays to impress potential mates (Boughman, 2002), cephalopods are masters at adapting camouflage colouration to avoid predation (Widder, 2010), and frogs can display vibrant colours as warning signals (Cibulkova, Vesely & Fuchs, 2014). Photoreceptor cells located in the retina, called the cones and rods, are responsible for processing visual information (Kramer & Davenport, 2015). Rods are mostly utilised in low light conditions as there is only one light-detection pigment, and it allows for the visualisation of movement and shapes—these are most often used at night by nocturnal animals (Bartol & Musick, 2001). The cones facilitate colour vision via multiple pigments with varying peak absorbance that function best during high light conditions (Jacobs & Rowe, 2004). Furthermore, evolutionary history plays an important role on the ratio of rods and cones, and how this can change the organism’s visual capabilities (Schaeffel & Feldkaemper, 2016). For example, snake species that are predominantly active at night have only rod-like photoreceptors within their retina, in comparison, non-nocturnal species have only cone photoreceptors (Sillman et al., 1997). Moreover, some organisms possess additional photoreceptor organelles, known as oil droplets, which filter light rays before the cone processes it. This reduces some overlap between spectral sensitivities, and shifts the visual range towards longer wavelengths, allowing more colours to be visible within the organism’s visual spectrum (Granda & O’Shea, 1971; Vorobyev, 2003).

Green turtles (*Chelonia mydas*) have historically been the subject of microspectrophotometry and flicker electroretinography experiments to determine their spectral sensitivities. It was found that green turtle cones have peak absorbance between 325–400 nm, 440 nm, 502–515 nm, and 560–565 nm (Witherington & Bjorndal, 1991; Levenson et al., 2004; Mathger, Litherland & Fritsches, 2007; Schuyler et al., 2014). These results indicate that green turtles may see within the ultraviolet, blue–green, green, and green–yellow visual ranges (Braun et al., 2014). In addition to the four peak absorbances, green turtles also have four different oil droplet colours: fluorescent clear, non-fluorescent clear, red/orange, and yellow (Mathger, Litherland & Fritsches, 2007).

A behavioural response to varied colour stimuli suggests colour vision for that organism (Young, Salmon & Forward, 2012). Mrosovsky & Carr (1967) investigated green turtle (*Chelonia mydas*) hatchling colour vision by assessing their attraction to blue, green, and red lights of varying intensities. It was concluded that blue was the most attractive, and red the least attractive (Mrosovsky & Carr, 1967). Moreover, Mrosovsky & Shettleworth (1968) conducted a similar experiment, and described the same conclusions (Mrosovsky & Shettleworth, 1968). Lastly, Witherington & Bjorndal (1991) updated the methodology and found again that green turtle hatchlings showed an increased preference for the shorter wavelength stimuli, specifically 360 nm to 500 nm, colours in the UV to blue visual range (Witherington & Bjorndal, 1991). These three studies used light of varied intensity to assess colour attraction within a terrestrial platform.

Colour changes as a result of the surrounding environment because it is a function of natural light from the sun (Jacobs, 1992). The energy from light is converted into electrical energy by the outer segments of the photoreceptors, the ganglion cells next transmit this information to the brain via chemical and electrical synapses, and vision occurs (Twig, Levy & Perlman, 2003). Furthermore, light penetrates at different rates depending on the medium, for example water is much denser than air, and as a result the light cannot penetrate as deep (Gislen & Gislen, 2004). Other factors such as the concentration of phytoplankton within the water column, and the quantity of suspended organic matter will also decrease the depth in which light can penetrate (Abdelrhman, 2016). Longer wavelengths (red) are absorbed near the surface of the water, and shorter wavelengths (blue) are scattered and visible below greater depths (Widder, 2010). Therefore, objects that appear red at the surface of the water or within the air will not appear red below certain depths. The availability of light within an environment can also have an impact on the inhabitant’s evolution. Interestingly, human children are less likely to exhibit myopia or short-sightedness if they spend long periods of time outdoors (Schaeffel & Feldkaemper, 2016). Saltwater turtles have vitamin A_1_-derived chromophores which result in more blue-shifted pigments, compared to freshwater turtles whom have vitamin A_2_-derived chromophores resulting in more red-shifted pigments. Freshwater systems are on average shallower, and would have a higher abundance of longer wavelengths available. This is compared to saltwater systems that have great depths with only shorter wavelength light available deep below the surface. This evolution for either vitamins A_1_ or A_2_, in relation to living within freshwater or saltwater environments, can also be seen in fish species (Liebman & Granda, 1971; Beatty, 1984; Enright et al., 2015; Emerling, 2016). Moreover, an eye adapted to only aquatic vision is emmetropic when submerged, and myopic within the terrestrial environment, resulting in focused vision under water and unfocused vision within the air (Land, 1990). For example, the eye of a terrestrial bird relies on the cornea and lens to refract light into the retina for vision. When submerged the refractive power of the cornea is minimal as the refractive index of water and the aqueous humor are too similar, therefore, the lens becomes more responsible for refracting light into the retina (Howland & Sivak, 1984). The Adélie penguin (*Pygoscelis adeliae*) is amphibious in nature; hunting for food under water, and utilising land for travel, moulting and breeding (Oliver et al., 2013), therefore, focused vision is essential in both an aquatic and terrestrial environment. Their cornea evolved to be abnormally flatter and the lens is significantly more spherical when compared to other birds (Sivik & Vrablic, 1979). It is suggested that these adaptations facilitate more focussed vision when submerged. Similar to Adélie penguins, green turtles feed under water and utilise the land for nesting. They possess a rounded lens, similar to many fish species, which compensates for the reduced corneal refraction capabilities when fully submerged (Ehrenfeld & Koch, 1967). Therefore, vision within both the terrestrial and aquatic environments is likely to be focussed.

Green turtles utilise terrestrial vision when breathing, nesting, and during their maiden journey into the ocean as a hatchling (Frick, 1976), and aquatic vision for foraging, mating, and navigating their immediate environment. It has been suggested that hatchlings are attracted to shorter wavelengths within the blue visual range when tested in a terrestrial environment, and are behaviourally attracted towards it (Mrosovsky & Carr, 1967; Mrosovsky & Shettleworth, 1968; Witherington & Bjorndal, 1991). Green turtles spend the majority of their life in the water, therefore questions around their colour vision within this different environment need to be investigated. Do green turtles continue to behaviourally respond to blue stimuli when under water? This current study was designed to understand green turtle hatchling’s selections of varied colour stimuli under water. The investigation determined whether the attraction towards blue continued when the animal is fully submerged under water, as well as determining whether colour saturation of the stimuli influences colour discrimination, and whether housing animals in different coloured tanks influences their visual choices.

## Materials and Methods

### Research animals and research facility

Forty-one newly emerged green turtle hatchlings from the same nest were collected from Heron Island, Queensland (23°26′S, 151°51′E) under permits from the Department of Environment and Heritage Protection (WITK15765815), Great Barrier Reef Marine Park Authority (G13/35955.1), and James Cook University Animal Ethics (A2309). The hatchlings were individually housed at the James Cook University Turtle Health Research Facility, The Caraplace, in 50 L rectangular tubs maintained at 26 °C ± 1 °C, with six separate tubs connected to the same salt water re-circulation system. Different coloured rearing tanks (two red systems, two white systems, two blue systems and one grey system) allowed for system and equipment colour coding to ensure biosecurity regulations. At the time of experimentation the turtles were six months of age, and the average weight was 106 g, ranging between 75 g and 138 g. Turtles were fed a diet of 5% body weight per day of gelatine cubes containing blended human grade fish, prawns, vegetables, fish pellets, and Sea Tabs^®^ Antioxidant Vitamins. Once blended, the food was light brown in colouration.

Light conditions of the facility were assessed using a Hioki 3423 Lux HiTESTER. The facility has solid walls, and gridded windows near the roof which allowed natural light to enter the room during daylight hours. The experimental tanks were never in direct sunlight. On all experimental days the weather was bright and sunny, and experiments were carried out within the same daylight hours. In addition to natural light, artificial overhead lighting was supplied by LED 2900–840 ET. All lights were switched on for the duration of the experiment, and the direct luminosity from these lights was an average of 14,453lx. These particular LEDs have a Colour Rendering Index (CRI) of 80, the CRI is a scale between 0 and 100 where the larger the number, the truer the colour. A CRI of 80+ is considered high quality lighting resulting in good quality colouration. Furthermore, the colour temperature of these lights was 4,000 K, resulting in white light colouration which contains all wavelengths but peaks in the blue range (Regency Lighting, 2015). Turtle holding tanks are solid, thick plastic tubs, therefore, no light could pass through them in any direction; all light came from above.

### Colour stimuli

Three colours were selected for experimentation: blue, yellow, and red. These colours were selected to represent the shorter wavelengths approximately 450–490 nm (blue), middle-length wavelengths between 560–590 nm (yellow), and longer wavelengths at 635–700 nm (red). The colour plates were created under the Red Green Blue (RGB) colour spectrum where each of the three variables are given a value between 0 and 255, and by altering these values a vast array of pigments can be created (Liu, Ling & Huang, 2011). Four different shades of each colour were created to represent varying levels of colour saturation, therefore, 12 different chromatic stimuli were generated. The saturation values were quantified by finding 100, 75, 50, and 25% of the RGB values for blue, yellow, and red (Table 1). Each colour plate was printed at Officeworks^®^ on a Fuji Xerox Colour C70 printer, and laminated to maintain waterproofing throughout the duration of each experiment. The overall size of the coloured central square was 100 mm × 100 mm. Ceramic Matt White Thaicera™ tiles 47 mm × 47 mm were glued to the back of the coloured plate to add weight. Printed and laminated colour plates were examined and visually matched to colours from The Munsell Book of Colour, an internationally utilised tool that allows consistency for the discussion of colours within the field (Munsell Colour Company, 1970) (Table 1). This practise is an international standard when communicating colour within the scientific community (Luke, 1992).

**Table 1:** A breakdown of the twelve experimental colours. The physical parameters of the colours used in the food choice experiments. The RGB values refer to the quantity of red, green, and blue within the colour. The Munsell numbers refer to the hue, value, and chroma according to The Munsell Book of Color where PB, purple blue; Y, yellow; and R, red (Munsell Colour Company, 1970).

Saturation percentages	Blue		Yellow		Red	
	RGB	Munsell	RGB	Munsell	RGB	Munsell
100	0, 0, 255	8.0PB 3.5/12	255, 255, 0	10Y 8/12	255, 0, 0	7.5R 4/16
75	0, 0, 191	7.5PB 3/12	191, 191, 0	10Y 7/10	191, 0, 0	7.5R 4/15
50	0, 0, 127	7.5PB 2/10	127, 127, 0	10Y 5/4	127, 0, 0	5R 3/10
25	0, 0, 64	7.5PB 2/3	64, 64, 0	10Y 3/4	64, 0, 0	5R 2/2

### Experimental design

The experiment was conducted during the hours of 10.00am and 1.00pm in August and September 2016 over nine days. Turtles were left to rest at least one day between tests, resulting in four experimental days overall. All experiments involving the same saturation treatment were conducted on the same day, with the order of saturation randomly determined. 100% colour saturation on the first day of the experimental period, 50% on the third day, 25% on the sixth day, and 75% on the ninth day. Each of the six possible arrangements of colour plates from left to right (BYR, BRY, YBR, YRB, RYB, and RBY) were allocated randomly to each system without replacement to ensure that each possible colour arrangement was used once for the grey system with six turtles, or twice for the red, white, and blue systems with twelve turtles. Therefore, each time an individual turtle was tested, it was with a different arrangement to avoid a left/right bias.

**Figure 1:**
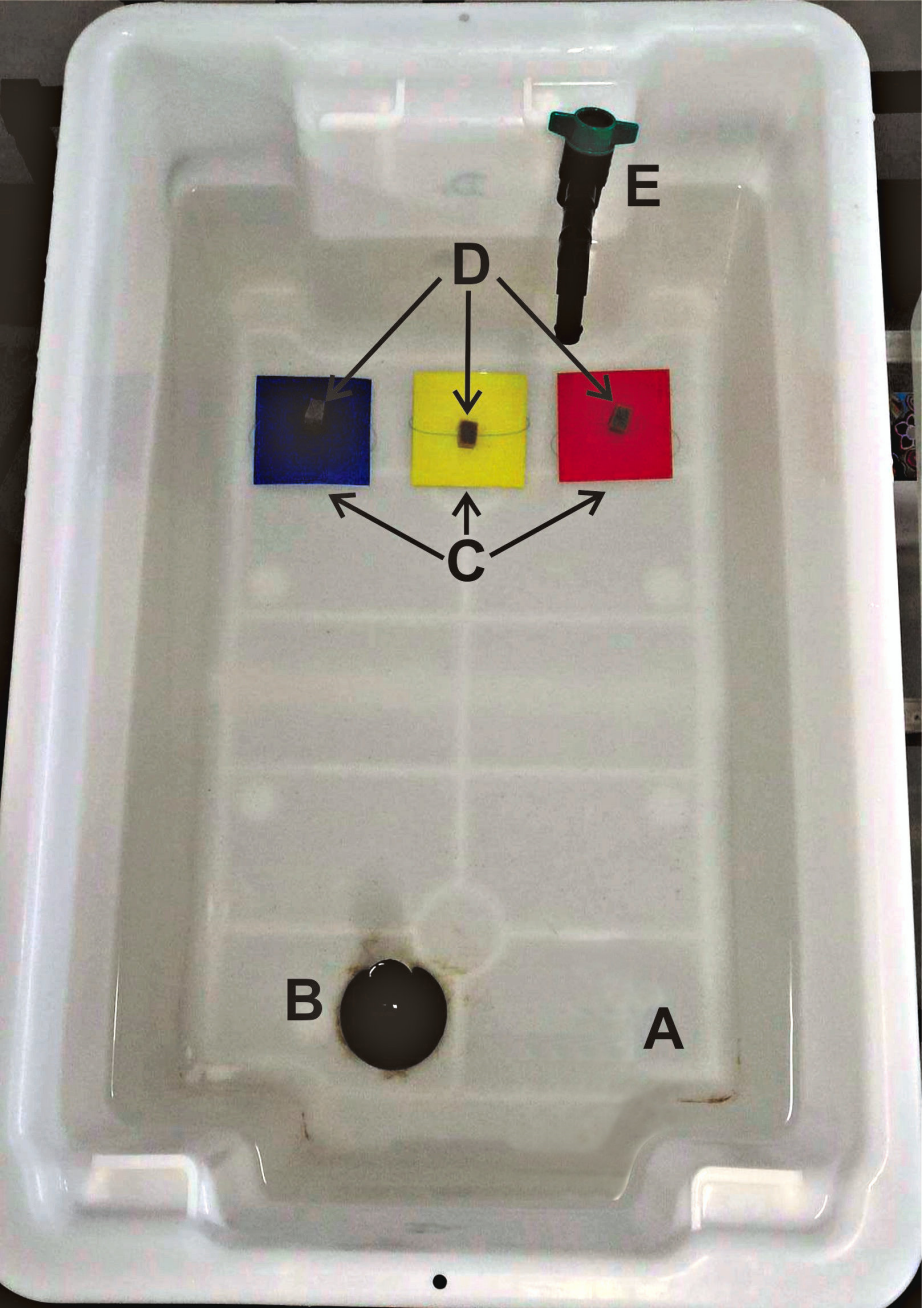
Experimental design of the food/colour choice experiment. (A) introduction point of the turtle, (B) outflow standpipe, (C) coloured plates, (D) food attached with fishing line and masking tape, (E) inflow water pipe. All four rearing and experimental tanks (red, white, blue and grey) had the same physical layout. Photo credit: Rebecca Jehne Hall.

A GoPro HERO4^®^ was mounted above the experimental tanks, and had the following settings applied: record at 1080 resolution, 60 FPS, narrow lens, and all lights and sounds disabled. Turtles were removed from their housing tanks, and kept in white ice-cream containers while the colour plates were organised, this process was at maximum two minutes. One 1 cm^3^ gelatine cube of food was threaded onto fishing line and fastened onto the ceramic tile with masking tape. A total of three colour plates (blue, yellow and red) representing one level of saturation (100, 75, 50, or 25%) was fitted with food and evenly spaced on the floor of each individual turtle’s housing tank (Fig. 1), thereby providing a three-choice selection for each test. Once the plates were arranged the turtle was reintroduced at the opposite end, held for three seconds, and then released. The test was concluded once a colour plate had been approached and the food had been bitten, or a ten-minute time limit was reached where no food was approached. The colour of the plate containing the food first engaged by the turtle was noted. Equipment was disinfected between trials. To reduce the possibility of distractions, the researchers did not directly watch the turtles during the trials. The video footage from the GoPro HERO4^®^ was broadcast onto an ASUS ZenFone 2^®^ via Bluetooth^®^, and was watched in real time with data recorded at a later date. The depth of water within the tanks was approximately 20 cm ± 5 cm, therefore it would not be filtering out specific wavelengths of colour, and it is reasonable to assume that the turtle would be seeing the same shades of colour within air and water due to the same amount of light available. This experiment aimed to assess whether the attraction towards blue was still apparent within an aquatic environment where vison may be different due to anatomical adaptions, as opposed to testing whether colours are different above and below water with this specific set up.

### Statistical analysis

The relationships between choice (the colour of the coloured plate), tank colour, saturation, and coloured plate arrangement was analysed with a multinomial logistic regression using the *multinom* function of R in RStudio^®^ in the form of:

\begin{eqnarray*}\text{CHOICE}\sim \text{TANK}\ast \text{SATURATION}+\text{ARRANGEMENT} \end{eqnarray*}

**Figure 2:**
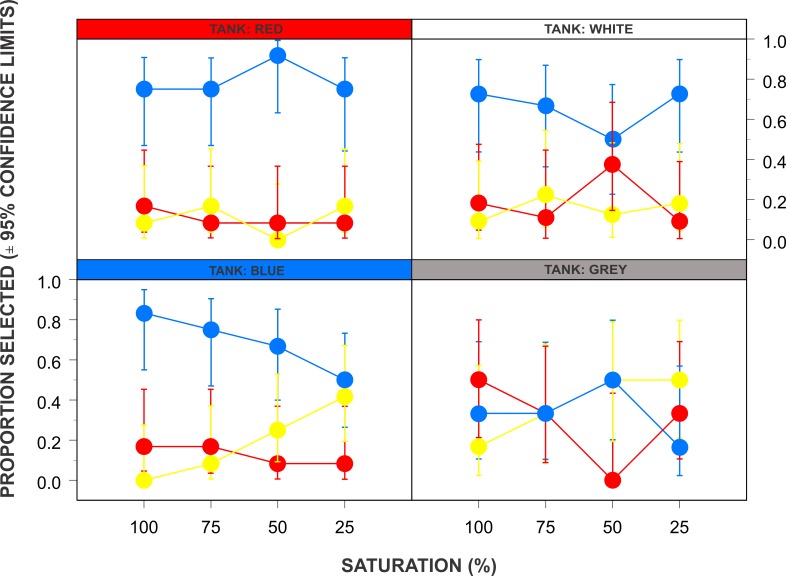
The relationships between tank colour, background colour and shading on food choice in the sea turtle *Chelonia mydas.* The probability of a hatchling green turtle (*Chelonia mydas*) selecting food on a particular coloured plate (blue, yellow or red) in relation to the stimuli saturation (100, 75, 50, or 25%) and rearing tank colour (red, white, blue or grey). Symbol colours match that of the chosen coloured plate.

Ninety-five percent confidence limits for the proportions with which each colour was selected (as a function of tank colour and stimuli colour saturation) were generated using the Agresti-Coull approximation (Jones et al., 2012), and graphed with S-Plus 8.0^®^ (Fig. 2).

## Results

There was six turtles reared in grey tanks, eleven reared in white tanks, twelve reared in red tanks, and twelve reared in blue tanks, totalling 41 individual turtles. Each turtle was exposed to three different target colours (blue, yellow, and red) per experiment, and one of the four different saturation rates (100, 75, 50, 25%) once each, resulting in a possible 164 data points. However, on five instances there was either a malfunction with the video recording or the turtle did not eat within the allotted 10-minute time frame, therefore 159 data points were analysed.

### Qualitative observations

Most turtles approached a colour plate and began eating food within one minute, only a small percentage (1.22% of the total data set) reached the ten-minute time limit and did not approach anything, and this only occurred twice with two different turtles. Upon entry into the tank, the turtles could be seen assessing which food to consume by looking at each plate for several seconds before making the choice, and swimming towards one colour plate. This behaviour was consistent regardless of the saturation of colour being tested.

### Quantitative observations

Tank colour had a significant effect on which colour food plate was selected (multinominal logistic regression, LR chi-square = 30.27, *df* = 9, *p* < 0.001, Table 2). As an overarching statement, turtles preferred food on blue plates (66.1% blue, 18.2% yellow and 15.7% red, respectively), however, this preference for blue targets seemed to be limited only to those turtles raised and tested in red (79.2%), white (66.67%) and blue tanks (68.7%). The colour preferences of turtles raised and tested in grey tanks were blue (33.3%), yellow (37.5%), and red (29.2%), respectively (Fig. 2). Colour saturation of the coloured stimuli had no significant effect on which colour plate was selected (LR chi-square = 7.61, *df* = 9, *p* = 0.055, Table 2), and there was no significant interaction between tank colour and saturation (LR chi-square = 4.32, *df* = 9, *p* = 0.889, Table 2). There was no significant effect with the arrangement of the three food plates in the tanks (LR chi-square = 21.32, *df* = 15, *p* = 0.127, Table 2).

**Table 2:** The statistical output aimed at investigating the relationships between target colours (colour of the tile that the food was on), tank colour, saturation, and stimuli arrangement. The statistical output of the multinomial logistic regression investigating the relationships between tank colour, saturation and stimuli arrangement on the likelihood that one particular coloured tile was selected. The colour of the tank refers to the tank that the turtles were reared in, and where the individual experiment occurred (blue, grey, white, or red). The saturation refers to the shade of colour for the stimuli (100, 75, 50, or 25%), and the arrangement refers to how the stimuli were organised from left to right (BYR, BRY, YBR, YRB, RBY, RYB).

Variable	LR chi-square	*Df*	*P*-value
Tank colour	30.2718	9	0.0004
Saturation	7.6115	3	0.0548
Coloured tile arrangement	21.3232	15	0.1268
Tank colour and saturation	4.3204	9	0.8891

## Discussion

This study shows that green turtle hatchlings choose food from coloured plates in a non-random fashion, providing evidence that they can differentiate colours under water. Furthermore, testing multiple levels of colour saturation, and finding that food on the same coloured plate was consistently selected, indicates that green turtle hatchlings have a strong attraction towards food on a blue background.

The turtles were housed in different coloured tanks (red, white, blue, and grey) within The Caraplace. These turtles have never been in the ocean; their exposure to colour is limited to the equipment utilised within the facility in the form of their tank colours, cleaning utensils (which were colour coded to their tank colours), and clothing worn by volunteers and researchers. Upon initial entry into the facility, animals were randomly assigned a coloured tank. At the time of experimentation each turtle was six months of age, and had lived within their individual tank for that duration. The statistical analysis revealed that the tank colour had a significant influence on which colour plate the turtles selected. However, blue was selected in significantly more instances than the other choices of yellow and red, regardless of the colour of the tank. There is an exception within the grey tanks where no colour was selected significantly more than the others suggesting that the environment in which an organism is raised could have an effect on their visual capabilities.

Hu et al. (2011) raised guinea pigs in either violet, green, or white (control group) light facilities, and after eight weeks dissected the eyes to determine whether short-wavelength (S-cones) and medium-wavelength (M-cones) sensitive cones changed in density. They found that eyes reared in green light had a higher density of M-cones, and eyes reared in violet light had a higher density of S-cones, when compared to the control group (Hu et al., 2011). Their conclusions were that visual anatomy has developmental plasticity where animal’s eyes adapt to their specific environment over time. The green turtles in the current experiment that were raised in grey tanks showed less attraction towards blue compared to the turtles raised in different tank colours (Fig. 2). It is reasonable to assume that the eye of a turtle raised in a blue tank would express more short-wavelength (S-cones) cones, compared to those raised in the white, red, or grey tanks. This is due to developmental plasticity where the eye of the turtle becomes better adapted to the environment it lives it, much like the guinea pigs (Hu et al., 2011). As more S-cones are expressed, that would leave less room for M-cones or L-cones, thus their ability to discriminate colours in the medium-wavelength or long–wavelength ranges would be compromised. Similarly, it is reasonable to assume that turtles reared in a red tanks would have more L-cones. The results of this experiment show that turtles reared in blue tanks selected food on blue plates more frequently than the other options, however, it would then be expected that turtles reared in red tanks should select red targets more frequently, and this is not the case (Fig. 2). The selection of food on blue plates was highest in the red tanks with 79.17%, compared to 10.42% and 10.41% for yellow and red, respectively. It is possible that an innate attraction towards blue yielded a stronger behavioural response, thus blue was selected more than the animal’s usual surrounding environment. Interestingly, within the grey tanks there was not a more frequently selected coloured stimulus where blue (33.33%, *n* = 8), yellow (37.5%, *n* = 9), and red (29.17%, *n* = 7) all had similar rates of selection. Other organisms exhibit similar visual developmental plasticity, for example, goldfish reared in light and dark environments showed changes to the anatomical structure within the eye (Wagner, 1980), and chickens reared under bright-light and dim-light conditions showed different oil droplet pigmentation due to the varied lighting treatments (Hart, Lisney & Collin, 2006). As the attraction towards the blue stimulus is lower within the grey tanks, it is reasonable to assume that this tank specifically has another factor not controlled within this experiment. The grey colouration is quite dark providing a darker environment for the turtles, and perhaps providing less contrast between the background and the colour plates. It could be similar to the goldfish (Wagner, 1980) where rearing in a darker environment changes the eye structure, and presumably the visual capabilities. The findings from the current experiment support the findings of the Hu et al., Wagner, and Hart et al. that the environment in which an organism is raised will impact their visual capabilities. Understanding the effects of rearing green turtles in different coloured tanks requires further experimentation. Furthermore, achromatic cues associated with colour targets could potentially influence the choice of colours by individuals (Siniscalchi et al., 2017). While they found no evidence of achromatic cues influencing colour choices for domesticated dogs, this possibility cannot be excluded in the current turtle study.

Visual anatomy can change throughout an organism’s life according to their environment, but also their lifestyle needs. The brown trout (*Salmo trutta*) relocates from living in freshwater to within the ocean as the fish matures. During this habitat shift the optical anatomy also changes to better accommodate prey within the new environment (Bowmaker & Kunz, 1987). Green turtles also shift habitats and feeding habits as they age. Hatchlings drift within the pelagic environment feeding opportunistically on molluscs and crustaceans (Boyle & Limpus, 2008). Isotopic analysis of young green turtle’s scutes revealed that on average the animal spends the first three to five years feeding carnivorously within open water, before shifting diets towards a more herbivorous lifestyle within coastal waters (Reich, Bjorndal & Bolten, 2007). Schaeffel (1991) found that aquatic snakes that feed within the water column have evolved good under water vision, but aquatic snakes that do not hunt aquatic prey do not possess developed vision when submerged (Schaeffel, 1991). As green turtles feed on aquatic species regardless of their age, it can be assumed that they need to have well developed vision under water, and by extension colour vision. The turtles used within the current experiment were six months old. It is likely that acute vision and colour differentiation may be advantageous when foraging within a pelagic environment. Schuyler et al. investigated the colours of consumed plastics in the gut contents of green turtle hatchlings, and found that blue items were consumed the least. It was suggested that this be due to an inability to differentiate between the blue background of the open-ocean, and the blue objects (Schuyler et al., 2014). Findings from the current experiment show that hatchlings prefer food on a blue background. An ability to differentiate between colours is acute with hatchlings at this age, and perhaps the minimal amount of blue plastics in the gut contents was due to another factor, as opposed to an inability to find it. It is important to note however, that turtles in the current experiment were not attracted to blue food, but rather food on a blue background. Green turtles have oil droplets within their cones which shift spectral sensitivities towards longer wavelengths (Granda & O’Shea, 1971). The possession of oil droplets allows more colours to be available (Vorobyev, 2003) and as a result may assist in contrast between two objects of different colours (Mathger, Litherland & Fritsches, 2007). With reference to the current study, the red/orange and yellow oil droplets likely allowed better contrast between the yellow and red coloured plates, and the food. If this is the case, as the turtles continued to eat from the blue coloured plates, it further provides evidence that green turtle hatchlings are attracted to blue. Furthermore, the saturation of blue was not a significant variable, indicating that various shades of blue are more attractive compared to the alternative choices. It is possible that an attraction towards blue could assist with sea–finding behaviour, or potentially foraging efforts where the ability to find contrast between the blue background of the ocean, and prey items would be essential for survival.

Understanding colour vision in animals allows for previously unexplored species-specific traits to be utilised in a different way. In New Zealand, an endemic parrot, the Kea (*Nestor notabilis*), was decreasing in population due to the consumption of poisoned bait laid out to kill invasive species. An experiment was conducted to discover a colour that could act as a visual deterrent to prevent the bird from eating the bait, green was found to be the least attractive colour (Weser & Ross, 2012). The authors accepted that other variables (size, shape, smell etc.) should be accounted for as well, however, utilisation of an organism’s innate attractive or deterrent behaviour towards colours opens up specified protective management possibilities. This concept of bait modification has also been utilised within the marine environment where it was found that dying bait dark blue has significantly decreased seabird interaction within the longline fishing community (McNamara, Torre & Kaaialii, 1999). Furthermore, Southwood et al. (2008) discuss the colouration of chemiluminescent lightsticks in relation to reduced mortality of sea turtle bycatch during longline fishing expeditions, (Southwood et al., 2008) and Swimmer et al. (2005) investigated dying squid for loggerhead (*Caretta caretta*) and Kemp’s ridley turtles (*Lepidochelys kempii*) both within a captive environment and outside in the field (Swimmer et al., 2005). Green turtles (*Chelonia mydas*) have been listed as an endangered species on the International Union for the Conservation of Nature’s Red List (Seminoff& Southwest Fisheries Science Center, US, 2004) since 1982 with population trends continuing to decrease internationally (Cavallo et al., 2015). The survival rate is lowest for hatchlings where only approximately one out of every one thousand hatchlings survive into adulthood (Triessnig, Roetzer & Stachowitsch, 2012). Therefore, new management techniques must be explored to increase survivorship.

In conclusion, behavioural experiments conducted with hatchling green turtles have shown that this species can discriminate between blue, yellow, and red, and that the preference towards blue continues despite varied colour saturations. Also, that this preference is influenced by the tank colour in which they were raised and experimented. Evolving the ability to differentiate between background and foreground may assist in survival during this critical life stage by allowing more advanced foraging skills. Utilising the species’ innate behavioural response to different colours may lead to targeted strategies that could increase survivorship of green turtle hatchlings during this vulnerable life stage.

##  Supplemental Information

10.7717/peerj.5572/supp-1 Supplemental Information 1 Three green turtle hatchlings and their colour selections

10.7717/peerj.5572/supp-2 Data S1 Raw data information The raw data for the experiment commented on in detail. Trial one (100%), trial two (50%), trial three (25%), and trial four (75%).

## Additional Information and Declarations

### Field Study Permissions

The following information was supplied relating to field study approvals (i.e., approving body and any reference numbers): Field experiments were approved by the Department of Environment and Heritage Protection (WITK15765815) and Great Barrier Reef Marine Park Authority (G13/35955.1).

### Data Availability

The following information was supplied regarding data availability: The raw data are provided in a Supplemental File.
